# Supplementary material for: Mother brain is wired for social moments
Source: eLife. 2021 Mar 25;10:e59436. doi: 10.7554/eLife.59436 (PMC8026217; doi:10.7554/eLife.59436)
Supplement: Figure 1—figure supplement 1—source data 1. — BFincl is calculated using the Baws factor approach across all matched models. Two factor Bayesian repeated measures ANOVA (PBO- OT × Time). [file elife-59436-fig1-figsupp1-data1.docx]

| Effects | P (incl, data) | BF_incl_ |
| --- | --- | --- |
| *PBO-OT* | 0.005 | 62.62 |
| *Time* | 0.005 | 169.17 |
| *PBO-OT*×*Time* | 0.995 | 197.08 |
