# Supplementary material for: Mother brain is wired for social moments
Source: eLife. 2021 Mar 25;10:e59436. doi: 10.7554/eLife.59436 (PMC8026217; doi:10.7554/eLife.59436)
Supplement: Table 2—source data 1. — BFincl is calculated using the Baws factor approach across all matched models. [file elife-59436-table2-data1.docx]

| Effects | P (incl, data) | BF_incl_ |
| --- | --- | --- |
| *ROI* | 2.282e-6 | 1.338e+30 |
| *PBO-OT* | 7.410-7 | 0.052 |
| *Self-Other* | 2.613e-7 | 0.131 |
| *Maternal Condition* | 1.277-5 | 7.052 |
| *ROI× PBO-OT* | 0.0004 | 0.0004 |
| *ROI× Self-Other* | 1.00 | 3.312e+6 |
| *ROI× Maternal Condition* | 0.004 | 0.004 |
| *PBO-OT× Self-Other* | 0.122 | 0.139 |
| *PBO-OT× Maternal Condition* | 1.00 | 1.346e+6 |
| *Self-Other× Maternal Condition* | 0.032 | 0.033 |
| *ROI× PBO-OT× Self-Other* | 8.062e-8 | 0.002 |
| *ROI× PBO-OT× Maternal Condition* | 1.634-9 | 0.0009 |
| *ROI× Self-Other × Maternal Condition* | 1.268e-7 | 0.001 |
| *PBO-OT× Self-Other× Maternal Condition* | 0.0002 | 0.049 |
| *ROI× PBO-OT× Self-Other× Maternal Condition* | 1.420e-21 | 0.003 |
