# Supplementary material for: Mother brain is wired for social moments
Source: eLife. 2021 Mar 25;10:e59436. doi: 10.7554/eLife.59436 (PMC8026217; doi:10.7554/eLife.59436)
Supplement: Figure 5—source data 1. [file elife-59436-fig5-data1.docx]

| Effects | P (incl, data) | BF_incl_ |
| --- | --- | --- |
| *ROI* | 0.224 | 0.103 |
| *Self-Other* | 0.120 | 0.049 |
| *Maternal Condition* | 0.999 | 432.24 |
| *ROI× Self-Other* | 0.002 | 0.005 |
| *ROI× Maternal Condition* | 0.002 | 0.004 |
| *Self-Other × Maternal Condition* | 0.010 | 0.023 |
| *ROI× Self-Other × Maternal Condition* | 1.404e-10 | 2.528e-9 |
