# Supplementary material for: Mother brain is wired for social moments
Source: eLife. 2021 Mar 25;10:e59436. doi: 10.7554/eLife.59436 (PMC8026217; doi:10.7554/eLife.59436)
Supplement: Transparent reporting form [file elife-59436-transrepform.docx]

***eLife’s* transparent reporting form**

We encourage authors to provide detailed information *within their submission* to facilitate the interpretation and replication of experiments. Authors can upload supporting documentation to indicate the use of appropriate reporting guidelines for health-related research (see [EQUATOR Network](http://www.equator-network.org/%20)), life science research (see the [BioSharing Information Resource](https://biosharing.org/)), or the [ARRIVE guidelines](http://www.plosbiology.org/article/info:doi/10.1371/journal.pbio.1000412) for reporting work involving animal research. Where applicable, authors should refer to any relevant reporting standards documents in this form.

If you have any questions, please consult our Journal Policies and/or contact us: [editorial@elifesciences.org](mailto:editorial@elifesciences.org).

**Sample-size estimation**

- You should state whether an appropriate sample size was computed when the study was being designed
- You should state the statistical method of sample size computation and any required assumptions
- If no explicit power analysis was used, you should describe how you decided what sample (replicate) size (number) to use

Please outline where this information can be found within the submission (e.g., sections or figure legends), or explain why this information doesn’t apply to your submission:

Data was collected from 32 subjects. Sample size was decided based on previous published oxytocin administration imaging studies using similar placebo-controlled within subject design (Wigton R, Radua J, Allen P, Averbeck B, Meyer-Lindenberg A, McGuire P, Sukhi S, Fusar-Poli P. 2015. Neurophysiological effects of acute oxytocin administration: Systematic review and meta-analysis of placebo-controlled imaging studies. J Psychiatry Neurosci 40.)

**Replicates**

- You should report how often each experiment was performed
- You should include a definition of biological versus technical replication
- The data obtained should be provided and sufficient information should be provided to indicate the number of independent biological and/or technical replicates
- If you encountered any outliers, you should describe how these were handled
- Criteria for exclusion/inclusion of data should be clearly stated
- High-throughput sequence data should be uploaded before submission, with a private link for reviewers provided (these are available from both GEO and ArrayExpress)

Please outline where this information can be found within the submission (e.g., sections or figure legends), or explain why this information doesn’t apply to your submission:

In the recruitment phase, psychopathology during the perinatal period was an exclusion criteria. Of the 32 subjects that has completed two fmri scans, 23 were analyzed (72%). After examining the quality of the data 6 mothers were excluded due to excessive head movement artifacts (movements≥3mm). In additional 3 participants we identified unexplained noise in the signal, found by contrasting the visual conditions vs rest. All 9 subjects were removed before analysis of the experimental effects. For detailed description of recruitment and exclusion criteria please refer to Materials and Methods- participants in the manuscript.

**Statistical reporting**

- Statistical analysis methods should be described and justified
- Raw data should be presented in figures whenever informative to do so (typically when N per group is less than 10)
- For each experiment, you should identify the statistical tests used, exact values of N, definitions of center, methods of multiple test correction, and dispersion and precision measures (e.g., mean, median, SD, SEM, confidence intervals; and, for the major substantive results, a measure of effect size (e.g., Pearson's r, Cohen's d)
- Report exact p-values wherever possible alongside the summary statistics and 95% confidence intervals. These should be reported for all key questions and not only when the p-value is less than 0.05.

Please outline where this information can be found within the submission (e.g., sections or figure legends), or explain why this information doesn’t apply to your submission:

Detailed information regarding the statistical analyses and their results can be found in Results section and in Table 2 in the manuscript.

(For large datasets, or papers with a very large number of statistical tests, you may upload a single table file with tests, Ns, etc., with reference to sections in the manuscript.)

**Group allocation**

- Indicate how samples were allocated into experimental groups (in the case of clinical studies, please specify allocation to treatment method); if randomization was used, please also state if restricted randomization was applied
- Indicate if masking was used during group allocation, data collection and/or data analysis

Please outline where this information can be found within the submission (e.g., sections or figure legends), or explain why this information doesn’t apply to your submission:

Restricted randomization was used in the current placebo-controlled, double-blind, two-period crossover designed study, so that prior to the first scan half of the subjects administered oxytocin and half administered placebo. In addition and independently, during the first scan half of the subjects viewed Version 1 of the paradigm and half viewed Version 2. Two versions of the paradigm differ in video-clips order so that self-other and maternal conditions were counterbalanced. For more details see Materials and Methods- procedure section and Figure 7 in the manuscript.

**Additional data files (“source data”)**

- We encourage you to upload relevant additional data files, such as numerical data that are represented as a graph in a figure, or as a summary table
- Where provided, these should be in the most useful format, and they can be uploaded as “Source data” files linked to a main figure or table
- Include model definition files including the full list of parameters used
- Include code used for data analysis (e.g., R, MatLab)
- Avoid stating that data files are “available upon request”

We shared raw, subject by subject, anonymized brain data (fMRI); group level data (e.g. unthresholded group maps on MNI template) and raw subject by subject data from the ROI analysis (csv and JASP files). These files are uploaded to our OSF account (<https://osf.io/mszqj/?view_only=0daf10c02c984ead8929452edf44e550>). We believe that these measures will allow full transparency of the data.

Please indicate the figures or tables for which source data files have been provided:
